# Plasma Phospholipid Fatty Acids and Risk of Atrial Fibrillation: A Mendelian Randomization Study

**DOI:** 10.3390/nu11071651

**Published:** 2019-07-19

**Authors:** Shuai Yuan, Susanna C. Larsson

**Affiliations:** 1 Department of Surgical Sciences, Uppsala University, SE-751 85 Uppsala, Sweden; 2 Unit of Cardiovascular and Nutritional Epidemiology, Institute of Environmental Medicine, Karolinska Institutet, SE-171 77 Stockholm, Sweden

**Keywords:** atrial fibrillation, diet, fatty acids, Mendelian randomization

## Abstract

Available evidence on the associations of dietary and circulating levels of long-chain n-3 fatty acids, which have potential antiarrhythmic properties, and other fatty acids with atrial fibrillation is conflicting and limited. We conducted a Mendelian randomization study to assess the associations between plasma phospholipid fatty acid levels and atrial fibrillation. Summary-level data of atrial fibrillation were available from 65,446 cases and 522,744 non-cases included in the Atrial Fibrillation Consortium. Sixteen single-nucleotide polymorphisms associated with ten fatty acids at significance level of *p* < 5 × 10^−8^ were identified as instrumental variables from the hitherto largest genome-wide association studies for plasma fatty acids. The fixed-effects inverse-variance weighted method was used to assess the association of individual plasma fatty acids and atrial fibrillation risk. The random-effects inverse-variance weighted method, weighted median method, and Mendelian randomization (MR)-Egger method were employed as the sensitivity analyses. Genetic predisposition to higher levels of any of the ten individual fatty acids was not associated with atrial fibrillation risk.

## 1. Introduction 

Atrial fibrillation (AF) is a prevalent chronic arrhythmia in adults [1] and a risk factor for ischemic stroke [2], heart failure [3], and death [4]. A systematic review including 184 population-based studies, published from 1980 to 2010, estimated that the number of individuals diagnosed with AF was 33.5 million (20.9 million men and 12.6 million women) worldwide. In 2010, disease burden caused by AF, measured as disability-adjusted life-years, was 64.5 and 45.9 per 100,000 population in men and women, respectively, and increased by approximately 19% in both men and women from 1980 to 2010 [5]. Available therapies for AF have potentially detrimental health effects [6,7]. Thus, finding modifiable risk factors for AF is of great urgency. 

Fatty acids (FAs), a major part of a daily diet, may play a role in the development and recurrence of AF [8]. However, available epidemiological evidence on the associations of dietary or circulating fatty acids with risk of AF are conflicting and limited. In a randomized controlled trial, involving 6705 individuals, the risk of AF was reduced by 38% in the group following a Mediterranean diet with high extra-virgin olive oil intake compared with the control group [9]. Nevertheless, most but not all observational studies observed null associations of dietary and circulating n-3 polyunsaturated fatty acid with the risk of AF [10,11,12,13]. In a cohort study of 72,984 Swedish adults followed for 12 years, high n-3 polyunsaturated fatty acid intake was not associated with incident AF in men and women [10]. Studies of the associations of specific n-6 polyunsaturated fatty acids (PUFAs) and monounsaturated and saturated FAs with AF are scarce. The lack of association between FA intake and AF in most observational studies could potentially be related to misclassification of FA intake. Moreover, observational studies are prone to residual confounding and reverse causality, which can bias the results in an unpredictable direction. 

Mendelian randomization (MR) is an epidemiological approach of diminishing misclassification of exposure, residual confounding, and reverse causality by utilizing genetic variants as instrumental variables of exposures [14]. Genetic variants are randomly assorted at conception, thereby having no connections to self-adapted lifestyle factors and behaviors. Reverse causality is eliminated as disease cannot modify genotype. This method is based on instrumental variable analysis and can strengthen the inference on the causal nature of exposure-outcome associations. We conducted an MR study to assess the potential causal associations of ten plasma FAs with risk of AF.

## 2. Methods

### 2.1. Assumptions of MR Study and Study Design

**Figure 1:**
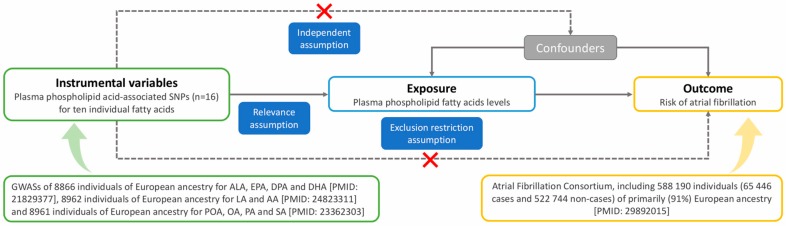
Schematic diagram of the Mendelian randomization assumptions underpinning a Mendelian randomization analysis of the association between plasma fatty acid (FA) levels and atrial fibrillation (AF). AA, arachidonic acid; ALA, α-linolenic acid; DHA, docosahexaenoic acid; DPA, docosapentaenoic acid; EPA, eicosapentaenoic acid; GWASs, genome-wide associations studies; LA, linoleic acid; OA, oleic acid; PA, palmitic acid; POA, palmitoleic acid; SA, stearic acid; SNPs, single-nucleotide polymorphisms.

There are three basic assumptions for an MR study, as shown in Figure 1 [15]. First, the genetic variants selected as instrumental variables should be robustly associated with the risk factor of interest (relevance assumption). Second, the used genetic variants should not be associated with potential confounders (independence assumption). Third, the genetic variants of an exposure should affect the risk of the outcome only through the risk factor, not via alternative pathways (exclusion restriction assumption). 

This MR study is based on summary-level data from four large published genome-wide associations studies (GWASs) on plasma fatty acids [16,17,18] and AF [19]. Detailed information on the GWASs, included studies, or consortium and single-nucleotide polymorphisms (SNPs) used as instrumental variables is presented in Figure 1 and Table 1. All studies included in the GWASs were approved by relevant institutional review boards and participants provided informed consent. The current MR analyses have been approved by the Swedish Ethical Review Authority.

### 2.2. Genetic Variants Selection

In this MR study, ten FAs were selected, including α-linolenic acid, eicosapentaenoic acid, docosapentaenoic acid, docosahexaenoic acid, linoleic acid, arachidonic acid, palmitoleic acid, oleic acid, palmitic acid, and stearic acid. In total, sixteen distinct SNPs associated with one or more of these FAs at a genome-wide significance threshold (*p* < 5 × 10^−8^) were identified from the largest available GWASs of plasma phospholipid FA levels, as shown in Figure 1 [16,17,18]. Seven single-nucleotide polymorphisms (SNPs) in four loci were identified to associate with n-3 PUFAs in a GWAS of 8866 individuals of European ancestry (one SNP for α-linolenic acid, one SNP for docosahexaenoic acid, two SNPs for eicosapentaenoic acid, and three SNPs for docosapentaenoic acid) [16]. Five SNPs in three loci were selected for n-6 PUFAs from 8962 European ancestry adults (two SNPs for arachidonic acid, three SNPs for linoleic acid) [17]. Five SNPs in four loci, one SNP, and four SNPs in three loci were selected for n-7 monounsaturated FAs, n-9 monounsaturated FAs and saturated FAs, respectively, from five prospective studies with 8961 individuals of European ancestry (one SNP for oleic acid, one SNP for palmitic acid, three SNPs for stearic acid, five SNPs for palmitoleic acid) [18]. All SNPs for each FA were in distinct gene regions and in linkage equilibrium. One standard deviation (SD) was scaled as the unit of change of FA levels in plasma because the concentrations of different FAs vary substantially. The SD values were obtained from the largest of the five cohorts included in the GWASs of FAs [20]. One SD change in the present study corresponded to 2.69, 1.96, 0.05, 0.30, 0.17, 0.89, 1.17, 0.18, 1.64, and 1.19 units in % total fatty acids for linoleic acid, arachidonic acid, α-linolenic acid, eicosapentaenoic acid, docosapentaenoic acid, docosahexaenoic acid, oleic acid, palmitoleic acid, palmitic acid, and stearic acid, respectively. Detailed information for each SNP is presented in Table 1. 

### 2.3. Data Source for AF

Summary-level data for AF were obtained from the Atrial Fibrillation Consortium’s 2018 dataset, which included 588,190 individuals of primarily (91%) European ancestry (65,446 cases and 522,744 non-cases) [19]. AF was defined as paroxysmal or permanent AF or atrial flutter. Detailed information of the association between each FA-associated SNP and AF is displayed in Table 1. 

### 2.4. Statistical Analysis

In the primary analyses, we report odds ratio (OR) estimates, with 95% confidence intervals (CI), of AF from the fixed-effects inverse-variance weighted method, which essentially combines the SNP-specific ratio estimates. Each ratio estimate was obtained by dividing the beta coefficient for the SNP-AF association with the corresponding estimate for the SNP-FA association. The ratio estimates were scaled per 1 SD increase in FA levels. For individual fatty acids with at least 3 SNPs, sensitivity analyses were performed, including the inverse-variance weighted method under the random-effects model, weighted median method, and MR-Egger regression method. Considering two strongly AF-associated SNPs in linoleic acid (rs10740118) and palmitoleic acid (rs603424), we performed leave-one-out analyses for these two FAs. The inverse-variance weighted method estimates the most precise associations but is sensitive to invalid instrumental variables with strong pleiotropic effects [21]. The weighted median approach can provide a consistent estimate under the condition that ≥50% of the weight in the analysis comes from valid instrumental variables [21]. The MR-Egger regression approach is able to identify and correct for directional pleiotropy but has low statistical power [22]. All odds ratios of AF were calculated to each SD increase in genetically predicted plasma FAs.

To explore whether the FA-related SNPs have pleiotropic associations with other phenotypes at a genome-wide significance level, we searched a database of human genotype-phenotype associations (PhenoScanner V2) [23]. Power calculation was based on the method proposed by Brion et al. [24]. Associations with *p* values < 0.005, correcting for 10 tests (ten FAs), were considered statistically significant and *p* values between 0.05 and 0.005 were treated as suggestive evidence of association. All *p* values were two-sided, and all statistical analyses were performed in Stata/SE 15.0 using the mrrobust package [25].

## 3. Results

We had over 85% power to detect an OR of 0.9 (or 1.1) of atrial fibrillation per one SD level change of eicosapentaenoic acid, docosapentaenoic acid, linoleic acid, oleic acid, arachidonic acid, palmitoleic acid, and stearic acid. However, we had only around 50% power for α-linolenic acid, docosahexaenoic acid, and palmitic acid to detect an OR of 0.9 (or 1.1), as shown in Table S1. There was a suggestive association between genetically predicted linoleic acid levels and AF in the fixed-effects inverse-variance weighted analysis. The odds ratio of AF per one SD increment in genetically predicted plasma levels of linoleic acid was 0.97 (95% confidence interval, 0.94, 0.99; *p* = 0.009), as shown in Figure 1. In sensitivity analyses, the significance did not remain. For one SD increment of linoleic acid levels, the odds ratio of AF was 0.97 (95% confidence interval, 0.86, 1.08; *p* = 0.55) in the random-effect inverse-variance model, with substantial heterogeneity between SNPs (*I*^2^ = 95%; *p* < 0.0001), and 0.98 (95% confidence interval, 0.95, 1.01; *p* = 0.13) in the weighted median analysis. No pleiotropy was detected in the MR-Egger analysis for linoleic acid (*p* = 0.22). In a leave-one-out analysis omitting rs10740118 (strongly associated with AF), the OR of AF per one SD increase of linoleic acid levels was 0.98 (95% CI, 0.96, 1.01; *p* = 0.13). 

**Figure 2:**
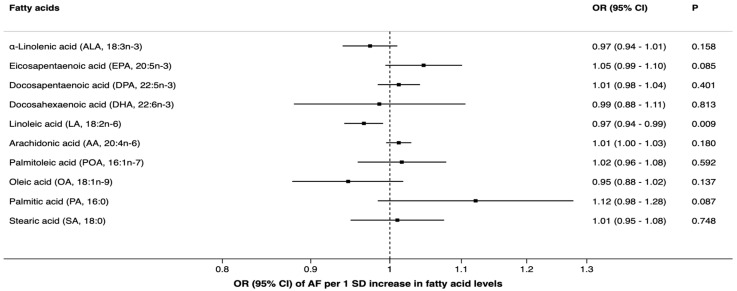
Associations of plasma fatty acid levels with risk of atrial fibrillation in Mendelian randomization analyses (fixed-effects inverse-variance weighted model). AF indicates atrial fibrillation; CI, confidence interval; OR, odds ratio.

Genetically predicted plasma levels of the other FAs were not associated with AF, as shown in Figure 2. Results of sensitivity analyses for docosapentaenoic acid, palmitoleic acid, and stearic acid were consistent with the main results, as shown in Table S2. In a leave-one-out analysis removing rs603424, which is associated with palmitoleic acid and also strongly with AF, the OR per one SD increase of palmitoleic acid was 0.94 (95% CI, 0.88, 1.01; *p* = 0.07). No significant pleiotropy was detected for docosapentaenoic acid, palmitoleic acid, and stearic acid, as shown in Table S2. As for potential pleiotropy, some of the FA-associated SNPs were associated with one or more phenotypes, including blood lipids, body mass index, height, alcohol intake, pulse rate, and certain immune cell counts, as shown in Table S3. 

## 4. Discussions and Conclusions

Findings of this MR study, along with results from experimental studies [26,27,28], suggest that plasma n-3 PUFA levels do not play a role in the development of AF. Moreover, the present study showed no associations of individual n-6 PUFAs or monounsaturated and saturated FAs with AF. 

Epidemiological data on the association of n-3 PUFA with AF have been conflicting. Most clinical experimental studies concluded that n-3 PUFA supplements had no effects on preventing incident AF among healthy individuals or either postoperative or recurrent AF among patients after cardiac surgery [26,29,30]. A systematic review of randomized controlled trials with 4677 patients showed that the OR of AF in the groups with n-3 PUFA supplementation was 0.95 (95% CI, 0.79, 1.13) for AF recurrence and 0.86 (95% CI, 0.71, 1.04) for postoperative AF, and strong heterogeneity between study results was detected [26]. Some observational studies have shown that high exposure to long-chain n-3 PUFAs decreases the risk of AF or AF recurrence [8,31], but a meta-analysis found no association between intake of long-chain n-3 PUFAs and AF risk [13]. Moreover, in a large-scale cohort of 72,984 Swedish adults with a 12 years follow-up period, dietary intake of n-3 PUFAs was not associated with a lower AF incidence after adjustment for other risk factors [10]. However, results from a Danish cohort study showed a higher risk of AF in men when total n-3 PUFAs replaced dietary saturated FAs [32]. The discrepancy among observational studies may be explained by residual confounding generated by AF-protective nutrients from diets rich in n-3 PUFAs or lifestyle behaviors of those with high PUFA intake. Dietary magnesium [33], certain vitamins [34], sedentary lifestyle [35], and obesity [14] have been found to be potential risk factors for AF. 

As for n-6 PUFAs, studies on its association with AF are limited. A Danish cohort study with 54,737 individuals (2274 incident AF cases) found no association between total n-6 PUFA intake and AF risk [36], which is in line with the present study. 

Available data on the association between saturated and monounsaturated FAs and risk of AF are inconsistent and sparse [37]. In the Cardiovascular Health study, high levels of circulating palmitic acid were associated with an increased risk of AF, whereas high levels of stearic acid were associated with a decreased risk of AF [37]. In a Danish cohort study, substitution of saturated FAs with monounsaturated, total, or n-6 PUFAs was not associated with risk of AF [32]. The present study based on genetics showed no association of specific saturated or monounsaturated FAs with AF. The reason for the disagreement is unclear but may potentially be related to residual confounding or misclassification of FA exposure in the cohort studies. 

A major strength is the large sample size for AF and the MR study design, which diminishes residual confounding and reverse causality that can bias the results from conventional observational studies. We confined the study population to individuals of European ancestry so that no population bias would influence the observed associations between individual plasma fatty acids and AF. A limitation of this study is that some SNPs were associated with more than one individual FA, which limited the possibility to disentangle the role of specific FAs for AF. Another shortcoming is that the statistical power to detect very weak associations (ORs between 0.9 and 1.1) was low for α-linolenic acid, docosahexaenoic acid, and palmitic acid. Thus, the associations of these FAs with AF need to be verified in larger causal inference studies, such as MR studies with more AF cases. In addition, certain included SNPs are associated with other risk factors for AF, such as body mass index and alcohol intake. The net effect of the changes in body mass index or alcohol intake caused by pleiotropic SNPs on atrial fibrillation risk is unknown. However, results of the MR-Egger analyses did not show any directional pleiotropy. Finally, three of the five cohorts included in the GWASs for plasma FAs were also included in the GWAS for AF that included over 50 studies. This sample overlap may have resulted in some bias in the causal estimates if the GWASs of FAs included cases of AF.

Genetic evidence from this study did not support any association of long-chain n-3 or n-6 PUFAs or monounsaturated and saturated FAs with AF risk. 

## Figures and Tables

**Table 1:** Characteristics of the single-nucleotide polymorphisms (SNPs) associated with plasma FA levels and their associations with atrial fibrillation.

Type of FA	FA	SNP	Nearby Gene	Chr	EA	NEA	% Variance Explained	Association with FA Levels			Association with AF		
								Beta *	SE	*p*	Beta ^†^	SE	*p*
n-3 PUFA	ALA	rs174547	*FADS1*	11	C	T	1.0	0.02	0.001	3.5 × 10^−64^	−0.011	0.008	0.158
n-3 PUFA	EPA	rs3798713	*ELOVL2*	6	C	G	0.4	0.04	0.005	1.9 × 10^−12^	0.002	0.008	0.797
n-3 PUFA	EPA	rs174538	*C11orf10*	11	G	A	1.7	0.08	0.005	5.4 × 10^−58^	0.014	0.008	0.073
n-3 PUFA	DPA	rs780094	*GCKR*	2	T	C	0.5	0.02	0.003	9.0 × 10^−9^	−0.008	0.007	0.295
n-3 PUFA	DPA	rs3734398	*ELOVL2*	6	C	T	2.7	0.04	0.003	9.7 × 10^−43^	−0.002	0.007	0.798
n-3 PUFA	DPA	rs174547	*FADS1*	11	T	C	8.4	0.08	0.003	3.8 × 10^−154^	0.011	0.008	0.158
n-3 PUFA	DHA	rs2236212	*ELOVL2*	6	G	C	0.7	0.11	0.014	1.3 × 10^−15^	−0.002	0.007	0.816
n-6 PUFA	LA	rs10740118	*JMJD1C*	10	G	C	0.2–0.7	0.25	0.050	8.1 × 10^−9^	−0.047	0.007	4.9 × 10^−11^
n-6 PUFA	LA	rs174547	*FADS1*	11	C	T	7.6–18.1	1.47	0.050	5.0 × 10^–274^	–0.011	0.008	0.158
n-6 PUFA	LA	rs16966952	*NTAN1*	16	G	A	0.5–2.5	0.35	0.040	1.2 × 10^–15^	–0.004	0.008	0.583
n-6 PUFA	AA	rs174547	*FADS1*	11	T	C	3.7–37.6	1.69	0.020	3.3 × 10^–971^	0.011	0.008	0.158
n-6 PUFA	AA	rs16966952	*NTAN1*	16	G	A	0.1–0.6	0.20	0.030	2.4 × 10^–10^	–0.004	0.008	0.583
n-7 MUFA	POA	rs780093	*GCKR*	2	T	C	0.2–0.9	0.02	0.003	9.8 × 10^–10^	–0.007	0.007	0.312
n-7 MUFA	POA	rs6722456	*RN7SKP93*	2	G	A	0.01–0.6	0.05	0.009	4.1 × 10^–8^	0.006	0.020	0.765
n-7 MUFA	POA	rs603424	*SCD/PKD2L1*	10	G	A	0.3–1.6	0.03	0.004	5.7 × 10^–15^	0.038	0.010	8.5 × 10^–5^
n-7 MUFA	POA	rs11190604	*HIF1AN*	10	G	A	0.02–0.7	0.02	0.004	5.7 × 10^–9^	−0.012	0.009	0.179
n-7 MUFA	POA	rs102275	*FADS1/2*	11	C	T	0.15–1.0	0.02	0.003	6.6 × 10^–13^	–0.011	0.007	0.136
n-9 MUFA	OA	rs102275	*FADS1/2*	11	C	T	0.3–2.1	0.23	0.020	2.2 × 10^–32^	–0.011	0.007	0.136
SFA	PA	rs2391388	*ALG14*	1	C	A	0.2–1.0	0.18	0.030	2.7 × 10^–11^	0.013	0.007	0.087
SFA	SA	rs6675668	*ALG14*	1	G	T	0.4–1.4	0.17	0.020	2.2 × 10^–18^	–0.011	0.007	0.121
SFA	SA	rs11119805	*LPGAT1*	1	T	A	0.01–0.7	0.17	0.030	2.8 × 10^–9^	0.008	0.011	0.435
SFA	SA	rs102275	*FADS1/2*	11	T	C	0.3–1.2	0.18	0.020	1.3 × 10^–20^	0.011	0.007	0.136

AA indicates arachidonic acid; AF, atrial fibrillation; ALA, α-linolenic acid; Chr, chromosome; DHA, docosahexaenoic acid; DPA, docosapentaenoic acid; EA, effect allele; EPA, eicosapentaenoic acid; FA, fatty acid; LA, linoleic acid; MUFA, monounsaturated fatty acid; NEA, none-effect allele; OA, oleic acid; PA, palmitic acid; POA, palmitoleic acid; PUFA, polyunsaturated fatty acid; SA, stearic acid; SE, standard error; SFA, saturated fatty acid; SNP, single–nucleotide polymorphisms. * The beta coefficients represent the change in percentage of total plasma fatty acid levels for each additional effect allele. ^†^ The beta coefficients represent the log odds ratio of atrial fibrillation for each additional effect allele.

## Supplementary Materials

The following are available online at https://www.mdpi.com/2072-6643/11/7/1651/s1, Table S1: Power calculation for the associations of plasma fatty acids with atrial fibrillation, Table S2: Sensitivity analyses for the associations of plasma fatty acids with atrial fibrillation, Table S3: Related traits of the single-nucleotide polymorphisms associated with plasma fatty acid levels from PhenoScanner search.

## Abbreviations

AF	Atrial fibrillation
CI	Confidence interval
FA	Fatty acid
GWAS	Genome-wide association study
MR	Mendelian randomization
PUFA	Polyunsaturated FA
SD	Standard deviation
SNP	Single-nucleotide polymorphism
